# Analysis of challenges faced and the scientific content of a hybrid pediatric surgical conference arranged during the COVID-19 pandemic

**DOI:** 10.1186/s43159-021-00135-2

**Published:** 2021-10-06

**Authors:** Muhammad Haris Patel, Jamshed Akhtar, Syed Muhammad Raees Hussain Taqvi, Tayyaba Batool

**Affiliations:** Department of Paediatric Surgery, National Institute of Child Health, Jinnah Sindh Medical University, Rafiqui Shaheed Road, Karachi, 75510 Pakistan

**Keywords:** Annual scientific meeting, Education, Virtual conference, Hybrid scientific conference, COVID-19 pandemic

## Abstract

**Background:**

Scientific conferences which are considered as an important event for dissemination of research and related academic activities were badly affected during the COVID-19 pandemic. Virtual format for interaction was used as an alternative method to continue such academic discourse. However, this did not provide the same level of communication and interest as that of in-person meetings. With evolving knowledge about the COVID-19 pandemic especially its transmission, role of vaccine, and observing standard operating procedures (SOPs), fear among healthcare providers is mitigated to some extent. Keeping in mind the importance of scientific conferences in the context of sharing knowledge and its impact on the training of faculty members and postgraduate residents, a hybrid conference was planned by the national association of pediatric surgeons.

The purpose of this study was to retrospectively review the challenges faced during the organization of this conference as well as to analyze the pattern of registration, number of abstracts received, the gender of the participants and their status, region of the country they represented, type of presentation made, and scientific subject covered. SPSS version 22 was used for data entry. Descriptive and inferential statistics were used to present data. Chi square test was applied to find out the association between categorical variables and a *p* value < 0.05 was considered as significant.

**Results:**

A total of 170 pediatric surgeons and postgraduate residents participated from all over the country and abroad. Nearly half (47.1%) of the registrants were postgraduate residents. Most of the participants (90%) opted for in-person attendance. The venue was selected with a capacity to house more than double the number of registrants with provision of safe distance. Availability of face masks, gloves, and sanitizers was ensured by the organizers. Packed meal boxes were arranged and served at the venue site in an open place on the terrace. A total of 97 abstracts were accepted for presentation that included 57 (58.8%) long oral podium and 40 (41.2%) poster presentations. Most of the studies (*n*=48–49.4%) were related to the subject of gastroenterology including pancreatico-hepatobiliary system and spleen. Majority of the presenters were male (*p* = 0.046) and postgraduate residents (*p* = 0.001).

**Conclusion:**

It was possible to organize a hybrid annual medical conference where most of the participants preferred physical presence. A rich scientific program was made to cater the needs for pediatric surgical fraternity. Residents made attractive presentations. It was noted that physical presence during clinical conference produced effective communication and learning.

## Background

Scientific conferences are held to share the findings of research conducted with an ultimate aim to inform the clinical practices in order to improve health-related issues [1]. During the COVID-19 pandemic, these events were postponed but as it is now accepted that the pandemic is going to stay and shall be part of new normal, initially the virtual and later hybrid conferences were started [2]. The virtual technology helped in holding these conferences. The pattern and content was almost similar but without physical presence [3]. However, it could not provide the same level of satisfaction as that of traditional medical conferences. It is known that being together and socializing help build and strengthen professional as well personal relations [4]. In-person conferences thus remained the most desired format.

The national association of pediatric surgeons planned to hold its international conference in the month of March 2021; however, due to the emergence of the second wave of COVID-19 in late 2020, it was canceled. Many of the members of the association were of the view that with standard operating procedures it can still be held in a hybrid fashion with the choice of the participants to join either in-person or virtually. The faculty of pediatric surgery at our institution accepted the challenge and considered it as an opportunity to hold this conference. The purpose was to involve pediatric surgeons from within the country and abroad to share their experiences and remain abreast with the latest trends in the era of the COVID-19 pandemic as discussed in literature as well [5].

Setting an agenda for the conference is important so that participants find it interesting and motivate them to get involved [6]. In addition, sessions should be arranged keeping interest of the senior as well as junior faculty members upfront. Involvement of postgraduate residents is another aim and dedicated slots must be kept for them. The conference was planned keeping all the above objectives in mind. This study was conducted to analyze the arrangements made at the venue of conference during the COVID-19 pandemic according to the directive of health authorities, the scientific content, pattern of participation, and type of presentation by pediatric surgeons from within the country and abroad. This was expected to set the agenda for future conferences both at national and international levels.

## Methods

This was an analytical study in which records of the conference held were retrospectively reviewed. IERB approval was granted under exemption category (IERB No. 10/2021). The data available from conference-related electronic material including official e-mails, Google Forms, and WhatsApp communication were retrieved. Variables analyzed included the number of registrations made by faculty members and residents, number of abstracts submitted by participating pediatric surgical departments, the number of oral long podium and poster presentations, and subject of research. In addition, the number and subject of invited talks and panel discussion were noted. The logistics arranged and details of the scientific program finally made were also analyzed.

The data were entered into SPSS^(R)^ version 22, and descriptive and inferential statistics were used for presentation. Comparison was made between the number of registered faculty members and residents, number of presentations made from different provinces, and the subjects of presentations. Chi square test was applied to find out the statistical significance and a *p* value < 0.05 was considered as significant.

## Results

From the first information of the conference till the last date of the online registration only 18-days were given. No on-site registration was allowed. A total of 170 registrations were received. This included 90 (52.9%) faculty members and 80 (47.1%) postgraduate residents. Most of the participants (*n* = 153–90%) opted for physical attendance and only 17 (10%) participants attended virtually that included those from outside the country. The venue was chosen based upon the number of registration made ensuring adequate seating capacity to maintain physical distance between the participants. Face masks, gloves, and sanitizers were also provided by the organizers throughout the conference.

The scientific program included four pre-conference workshops on the subject of minimal invasive surgery, endourology, basic biostatistics and SPSS data entry, basic epidemiology, and medical errors and negligence for medical doctors and healthcare workers. Seven invited lectures were delivered by experts from Singapore, Canada, India, UAE, Italy, and Egypt through Zoom^(R)^ virtual link. There were four invited talks by national speakers and two panel discussions on the subjects of disorders of sex differentiation in collaboration with the Society of Obstetrics & Gynecology, Pediatric Endocrine Society, and on issues related to pediatric surgical education and training at undergraduate and postgraduate level with the participation of eminent medical educationists from the country. The virtual link to the whole of the conference was made available to all the members of the association through its website even for those who were not registered.

There were 107 (63%) male and 63 (37%) female participants. The number of registered participants from different provinces is given in Table 1. The maximum number of participations was from region 2 (*n* = 77–45.3%) which was the place of conference venue followed by region 1 (*n*=56–33.5%) which is the most populous part of the country. The details of scientific paper presentations from different regions are given in Table 2. The maximum number of presentations were made from region 2 (*n*=40–41.2%). The institution-wise break up showed that the children hospital of region 1 largest city ranked first with 28 presentations followed by the national children institute of region 2 (*n*=21). On analyzing the subject of scientific paper presentations, it was noted that the major area covered was gastroenterology including pancreatico-hepatobiliary system and spleen (*n*=48–49.4%). The details are given in Table 3.

**Table 1:** Pattern of registration

Region	Faculty registration (***n***=90–52.9%)		Sub-total	Resident registration (***n***=80–47.1%)		Sub-total	Total (***n*** %)
	Male (***n***)	Female (***n***)		Male (***n***)	Female (***n***)		
**Region 1**	22	02	24	15	17	32	56 (33.5)
**Region 2**	28	17	45	11	21	32	77 (45.3)
**Region 3**	09	Nil	09	09	02	11	20 (11.7)
**Region 4**	03	Nil	03	04	01	05	08 (4.7)
**Other regions**	02	Nil	02	Nil	Nil	Nil	02 (1.1)
**Overseas**	04	03	07	Nil	Nil	Nil	07 (4.1)
**Total**	**68**	**22**	**90**	**39**	**41**	**80**	**170**

**Table 2:** Distribution of scientific paper presentation

Region	Oral long podium presentation (***n***)	Poster presentation (***n***)	Total (***n*** %)
**Region 1**	20	17	37 (38.1)
**Region 2**	23	17	40 (41.2)
**Region 3**	08	06	14 (14.4)
**Region 4**	Nil	Nil	Nil
**Other regions**	3	Nil	03 (3.0)
**Overseas**	3	Nil	03 (3.0)
**Total**	**57 (58.8%)**	**40 (41.2%)**	**97 (100%)**

**Table 3:** Research subject of scientific paper presentation

Subject	Oral long podium ***n*** %	Poster ***n*** %	Total ***n*** %
**Gastroenterology including pancreatico-hepatobiliary system and spleen**	30 (52.6)	18 (45)	48 (49.4)
**Oncology**	Nil	07 (17.5)	07 (7.2)
**Genitourinary system**	08 (14.0)	06 (15)	14 (14.4)
**Thorax**	03 (5.2)	04 (10)	07 (7.2)
**Infectious diseases**	11 (19.2)	Nil	11 (11.3)
**Miscellaneous**	05 (8.7)	05 (12.5)	10 (10.3)
**Total**	57 (58.7)	40 (51.3)	97 (100)

Most of the participants who made scientific presentations either long-podium oral or posters were male (*p*=0.046) and mostly by postgraduate residents (*p*=0.001). No association was found between gender and type of scientific content (oral/poster – p=0.099) neither was any association found between the type of scientific content, the subject of gastroenterology, and others (*p*=0.023). Also, there was no association between the location where the scientific study was conducted and the subject of study (*p*=0.3).

## Discussion

The results of this review showed that conference attendees were enthusiastic for physical participation and showed great interest in registration and submitted a large number of abstracts for presentation. This reflected that during COVID-19 pandemic research-related activities continued in addition to surgical services that were allowed as per the directive of health authorities. There was a huge challenge for holding this conference as reported in the literature as well [7]. The time was short and pandemic-related SOPs had to be followed so as to prevent any risk of infection to the conference participants who were also advised to get vaccination done as it was offered free of cost to the healthcare workers in Pakistan at that time. It was possible to hold this conference in hybrid format though many international conferences like that of the British Association of Paediatric Surgeons the year 2021 was in virtual format as multiple restrictions were imposed by the UK government. This trend is gradually shifting towards physical format and few conferences later this year and in 2022 shall be according to the old pattern. Our experience shall be helpful for the organizers of other associations.

From the first information until the conference itself, the organizers had 36 days. The registration time frame was kept limited to 18 days so as to know well in time as to the number of participants. No provision of on-site registration was provided. This was the most important step that organizers believe in ensuring the smooth conduct of the physical conference. This helped in arranging a suitable venue with adequate seating capacity with a safe distance. Despite the limited time frame, a total of 170 registrations were received. The pediatric surgical community, for the most part, was enthusiastic and opted for physical participation. This could be attributed to a year-long travel restriction that confined people to their home cities. The conference provided an opportunity to the pediatric surgical community to travel and socialize as well. Most of the participants were male however this was not statistically significant. Similarly, no difference was found with regard to faculty and postgraduate residents’ participation. This showed that females and residents were equally represented in the event. This observation is different from what has been reported recently where it was found that a number of females were less than males [8].

A total of 116 scientific abstracts were submitted till the last date mentioned in the time frame. Out of these, 97 were approved for presentation under different categories. The presenters followed all the guidelines provided by the scientific committee [9]. The explicit guidelines are important so as to ensure quality presentation with time for questions and answers. It is related not only to oral podium presentations but to poster format as well. It was noted that a large number of posters were presented by the residents in an impressive manner highlighting the importance of this format both for education and knowledge sharing [10]. Residents made a large number of both oral and poster presentations. This was an important learning experience for them and is encouraged during the training. They diligently collected data and made effective presentations reflecting command on the subject. This adds to their portfolios and encourages more involvement in research-related activities. The American Council of Graduate Medical Education (ACGME) guidelines for scholarly activities, many other organizations, and degree-awarding bodies require that residents must participate and make presentations in medical conferences. This is expected to produce a high level of satisfaction with the residency training. Multiple challenges are reported; most being related to administrative setup that are considered as hurdles for such activities especially in an era of COVID-19 pandemic [11]. However, our observations are quite encouraging. It also reflected that residency training program directors across the country and hospital administration fully supported this activity.

Most of the scientific papers were from the subject of gastroenterology including pancreatico-hepatobiliary system (49.4%), followed by urology and minimally invasive surgery. There was less content from other specialties especially from oncology and COVID-19 related challenges. This reflects that pediatric surgeons have confined their practice to the abdomen, urology, and tumor oncology. There is a need of inviting all surgeons involved in rendering services to pediatric surgery from across specialties. This shall help in establishing trans-discipline specialties with more comprehensive surgical care for children, a goal of the Global Initiative for Children’s Surgery [12].

Many important observations were made by holding the conference in an era of the COVID-19 pandemic. Firstly, physical conferences are still opted by the participants. Though there is a definitive risk of contracting infection but with observing SOPs and following the latest guidelines of health authorities, this can be addressed. Choosing an adequate venue with seating capacity and open space for serving packed meal were important steps. Secondly, the residents equally participated and made a number of presentations thus fulfilled a mandatory requirement of their training program. Thirdly, it provided an opportunity to meet and greet plus developing social networking. By involving eminent pediatric surgeons using the virtual portal from many countries across the globe helped in further developing liaison and seeking opportunities for residents’ exchange programs and faculty development.

## Conclusion

The hybrid conference was held successfully on a short notice with a large number of in-person participation. A number of eminent international speakers made virtual presentations. It provided an effective platform to deliberate upon important pediatric surgery-related issues. A pattern of research showed dominance of gastroenterology and pancreatico-hepatobiliary conditions as preferential interest of the participants. Arranging a physical conference during the COVID-19 pandemic is a challenge but not impossible.

## Data Availability

Data can be requested from the corresponding author.
